# Processing of Communication Calls in Guinea Pig Auditory Cortex

**DOI:** 10.1371/journal.pone.0051646

**Published:** 2012-12-12

**Authors:** Jasmine M. S. Grimsley, Sharad J. Shanbhag, Alan R. Palmer, Mark N. Wallace

**Affiliations:** 1 Institute of Hearing Research, Medical Research Council, Nottingham, United Kingdom; 2 Department of Anatomy and Neurobiology, Northeast Ohio Medical University, Rootstown, Ohio, United States of America; University of Rennes 1, France

## Abstract

Vocal communication is an important aspect of guinea pig behaviour and a large contributor to their acoustic environment. We postulated that some cortical areas have distinctive roles in processing conspecific calls. In order to test this hypothesis we presented exemplars from all ten of their main adult vocalizations to urethane anesthetised animals while recording from each of the eight areas of the auditory cortex. We demonstrate that the primary area (AI) and three adjacent auditory belt areas contain many units that give isomorphic responses to vocalizations. These are the ventrorostral belt (VRB), the transitional belt area (T) that is ventral to AI and the small area (area S) that is rostral to AI. Area VRB has a denser representation of cells that are better at discriminating among calls by using either a rate code or a temporal code than any other area. Furthermore, 10% of VRB cells responded to communication calls but did not respond to stimuli such as clicks, broadband noise or pure tones. Area S has a sparse distribution of call responsive cells that showed excellent temporal locking, 31% of which selectively responded to a single call. AI responded well to all vocalizations and was much more responsive to vocalizations than the adjacent dorsocaudal core area. Areas VRB, AI and S contained units with the highest levels of mutual information about call stimuli. Area T also responded well to some calls but seems to be specialized for low sound levels. The two dorsal belt areas are comparatively unresponsive to vocalizations and contain little information about the calls. AI projects to areas S, VRB and T, so there may be both rostral and ventral pathways for processing vocalizations in the guinea pig.

## Introduction

One of the main puzzles concerning the auditory cortex is in understanding the function of the many separate auditory areas. Species such as the monkey and cat may have 12 or 13 areas while even the evolutionarily primitive hedgehog has two areas [1]. It is assumed that individual areas are associated with separate functions. Evidence of this has been provided by studying sound localization in cats [2] and voice recognition in monkeys [3]. However, no previous study has compared the sensitivity of all auditory cortical areas in a species to conspecific social vocalizations. The guinea pig is a widely used species for studying the auditory system and its cortical region has an intermediate level of complexity with eight [4] or nine cortical areas [5]. We have previously studied parameters such as interaural level difference sensitivity [6] and interaural timing difference sensitivity [7] involved in sound localization in the guinea pig core cortical areas, but our preliminary evidence suggested that the belt areas were not very sensitive to these parameters. However, there was some evidence that conspecific vocalizations would be useful in distinguishing different functional roles for the auditory belt areas [8], [9]. Guinea pigs, like other hystricomorph rodents [10], have around 10 different vocalizations, many of which are produced in specific behavioural contexts [11]. Thus in this study we have analysed the responses of all eight auditory cortical areas to ten exemplars of their vocalizations which were chosen to represent the complete range of calls.

**Figure 1:**
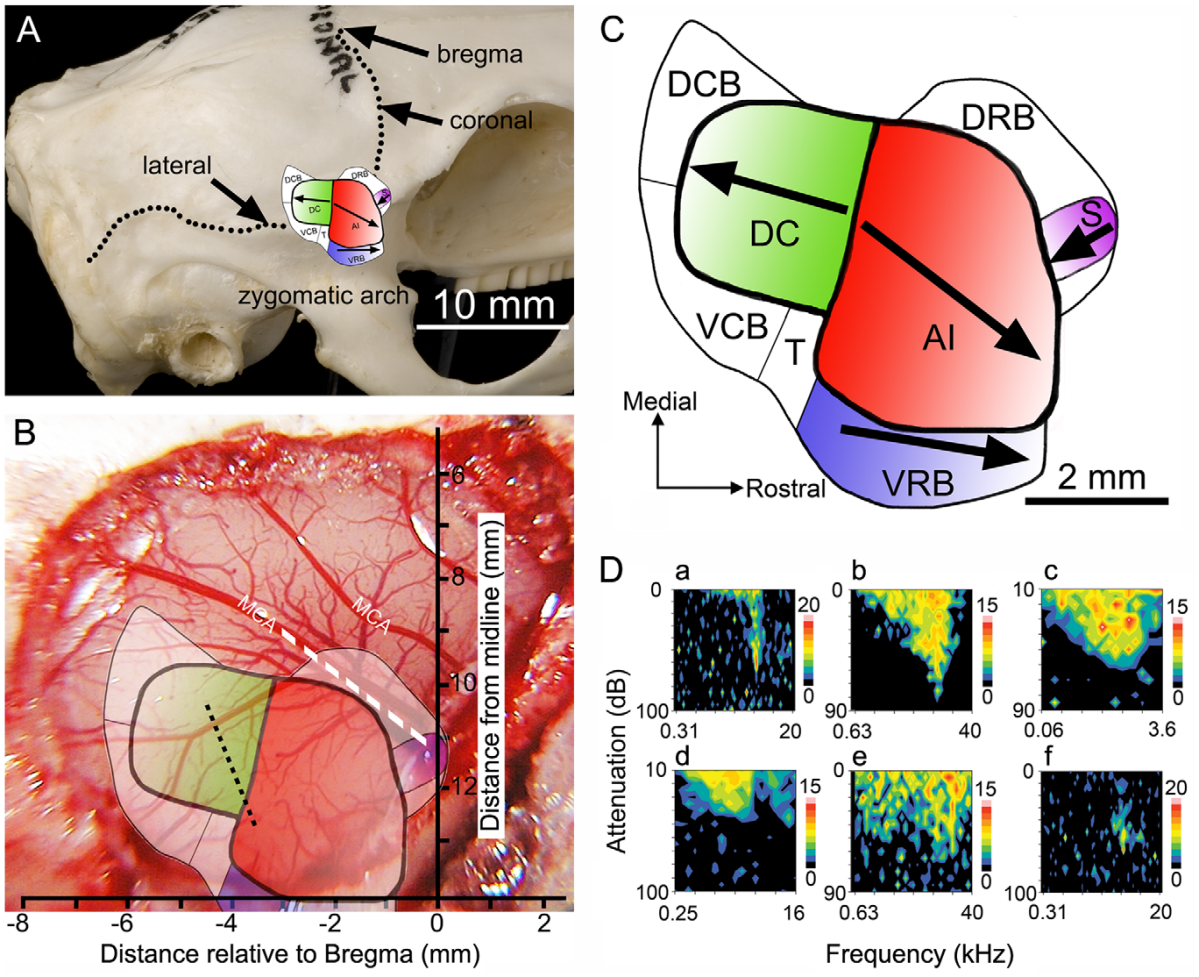
Tonotopic organization of auditory areas in relation to anatomical landmarks. **A** Lateral view of the guinea pig skull showing the approximate position of the auditory cortex in relation to surface landmarks. The core areas lie under the suture that forms the edge of the parietal bone just as it turns from being the coronal suture to forming the lateral edge. **B** Diagram showing approximate location of three auditory areas in relation to a large craniotomy. The position of the shallow pseudosylvian groove is indicated by the dashed white line and two branches of the middle cerebral artery are marked (MCA). The small field (area S) is located in the banks of this groove and lies about 11 mm from the midline as measured along the surface of the skull. The venous drainage of the auditory cortex forms a watershed indicated by the black dotted line and this coincides with the high-frequency border between AI and the area dorsocaudal to it (DC). The low-frequency border of AI is reliably found at about 1 mm behind bregma. The ventrorostral belt (VRB) area is covered entirely by the squamous temporal bone and is located just above the root of the zygomatic process. **C** Diagram of the eight auditory areas sampled in this study. The four areas with a tonotopic gradient have been shaded in with a colour gradient going from an intensely coloured high-frequency end to a pale, low-frequency end. The arrows also indicate the direction of the gradient from high-frequency to low-frequency. There are also four non-tonotopic areas that form part of the belt. These are the dorsorostral belt (DRB), dorsocaudal belt (DCB), ventrocaudal belt (VCB) and transition area (T). **D** Representative examples of the six main types of frequency response areas recorded among cortical units. A narrow; B “V” shaped; C broad; D double-peaked; E labile (non-tuned); F circumscribed. The results are plotted as temperature plots where the colour represents the number of spikes recorded in a 100 ms window during a single repetition of a randomly interleaved tone pip (100 ms duration).

In the guinea pig, the primary auditory area (AI) shares a high-frequency border with the other core area that is located dorsocaudal to it (DC) (Fig. 1C). Following on from the work of Redies et al. [12], we identified six belt areas (Fig. 1 A–C) by electrophysiological criteria: the ventrorostral belt (VRB), the transition area (T), the ventrocaudal belt (VCB), the dorsocaudal belt (DCB), the dorsorostral belt (DRB) and the small field (S) [4]. Four of the areas (AI, DC, VRB and area S) are tonotopically organized, which is useful in identifying their borders (Fig. 1C). Here, we compare the responses to a battery of communication calls across all areas of the auditory cortex to assess the relative contribution of each area to processing of these complex signals.

Auditory areas with a greater involvement in processing communication calls may show one or more of the following characteristics: (1) they may contain a greater proportion of cells that respond preferentially to communication calls over simple stimuli [13], [14], (2) they may contain many highly selective cells that only respond to one or very few out of a range of calls [15], [14], (3) they may contain a high proportion of discriminatory cells that respond to many calls but differentiate among them in their response patterns either by using a temporal code [16], [17] or (4) a rate code [18]. Finally, cortical areas may have other ways of representing information about a call [19], and they may use either a sparse or a dense representation [20].

## Methods

### Ethics Statement

All experiments were performed in accordance with the 1986 UK Animals (Scientific Procedures) Act and were conducted under project licence number 4003049 following approval by the University of Nottingham Ethics committee.

### Acoustic Stimuli

**Figure 2:**
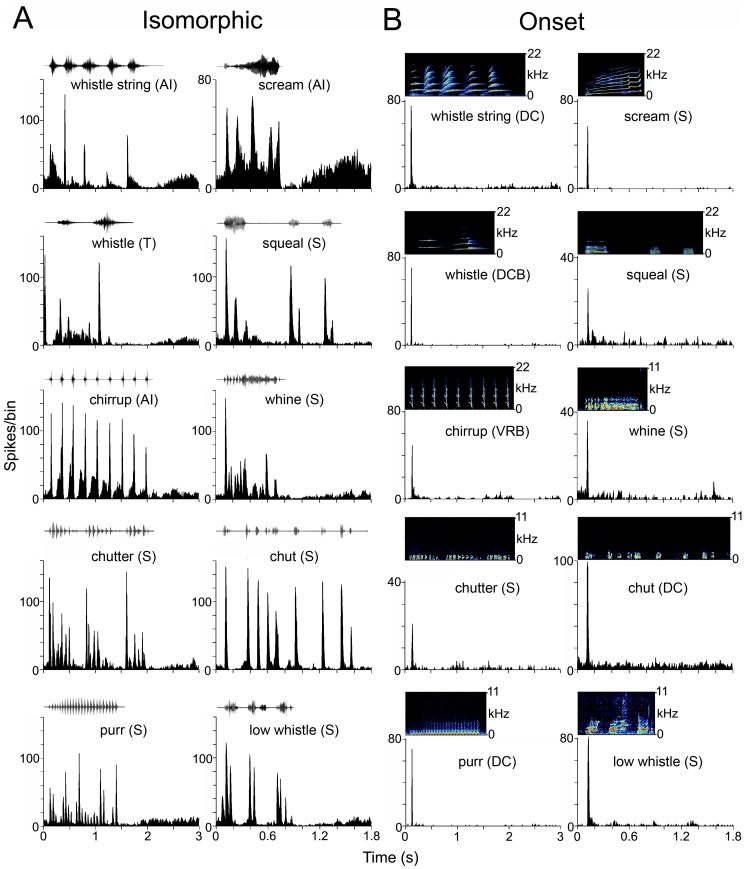
Spectrogram and waveform of the ten call exemplars used in this study and the resulting responses. A . The waveforms of the vocalizations and the responses to them of example neurons shown as PSTHs. The examples shown here all respond to all elements of the waveforms and hence produce a good representation (isomorphic) of the original waveform envelope. **B.** Spectrograms and PSTHs of the responses to the 10 vocalizations. Here all examples show neurons that only respond at the onset of the vocalization.

Recordings of vocalizations were made in a sound attenuating room containing two to four animals or from within the animal’s home cage in our own breeding colony. Vocalizations were recorded using a single-diaphragm condenser microphone (Model, B-5 Behringer), and the signal was passed via a mixer (Eurorack UB802) and a sound blaster (Creative, SBO 490) to a lap-top computer and stored using Adobe Audition 1 software (stereo, 24 bit float, 48.8 kHz sample rate). Recordings were made from adult animals over a period of five months until we had collected clear examples of all the main types of adult call identified by Berryman [11]. All calls are described using her nomenclature (see Fig. 2 and Fig. S1 for detailed spectrograms). These calls collectively contained the three basic elements present in mammalian calls: steady-state harmonically related frequencies, frequency modulations (both up and down) and noise bursts [20].

All the vocalizations used were tonal calls with a harmonic structure (Fig. 2 and Fig. S1). All of the calls contained low-frequency energy (<1 kHz) apart from the whistle, which was a constant frequency call with a fundamental of about 3 kHz. The rising whistle, which made up part of the whistle string, had a rising frequency ramp before the constant frequency part, while the chirrup was composed of a series of rapidly falling frequency glides, one of which is shown in Fig. 2. The other high-frequency call, the scream, is composed of a rising frequency glide, which in this example has a sudden frequency jump near the end. The squeal is mainly a constant frequency call with a fundamental of about 800 Hz, while the low whistle has frequencies that are modulated both up and down. The whine can also show small variations in frequency and has some separate bursts of sound at the start before the continuous segment. The chutter has short, low-frequency pulses of sound that are a bit shorter and more frequent than the pulses in the chut. The purr has the lowest fundamental (300 Hz) and has a very regular rhythmic structure.

The peak amplitude of all vocalizations were normalized and presented at a maximum sound level that was roughly equivalent to 80 dB SPL. Pure tones were generated by an array processor (Tucker-Davis Technologies AP2, Alachua, FL) at a 100 kHz sampling rate. Digital versions of 10 guinea pig communication calls were output via a digital-to-analogue converter and waveform reconstruction filters set at 1/4 the sampling rate (135 dB/octave elliptic: Kemo 1608/500/01 modules supported by custom electronics).

Auditory stimuli were delivered diotically through sealed acoustic systems, comprising modified Radio Shack 40–1377 tweeters joined via a conical section to a damped, 2.5 mm diameter probe tube that fitted into the speculum. The system was calibrated in each experiment using a Brüel and Kjær 4134 microphone with a 1 mm probe tube inserted close to the tympanic membrane and was flat ±10 dB to 30 kHz.

### Electrophysiological Methods

#### Surgical preparation

Recordings were made in 40 pigmented guinea pigs weighing 337–1007 g, some of which were also being used to collect data for separate studies. Surgical anesthesia was induced with urethane (4.5–5.5 ml/kg; 20% solution, i.p.) and supplemented as necessary by 0.1–0.2 ml Hypnorm (fentanyl citrate 0.315 mg/ml; fluanisone 10 mg/ml i.m., Janssen). Larger animals required proportionately less urethane. Anesthetic level was maintained at a level where the forepaw pinch reflex was just abolished by giving supplementary doses of Hypnorm about once an hour. Respiratory secretions were reduced by subcutaneous atropine sulfate and body temperature was maintained at 38°C by a rectal thermometer and heating blanket. The animals were artificially respired with 100% oxygen using a Harvard small animal ventilator model 683, and their end-tidal carbon dioxide levels were maintained between partial pressures of 28–38 mm of mercury. The animals were placed in a stereotaxic frame with hollow plastic speculae replacing the ear bars inside a sound-attenuating room. To prevent pressure building up in the middle ear that may interfere with sound perception, polyethylene tubing was inserted via small holes in the auditory bullae which were then resealed. A small incision was made in the dura of the posterior fossa to release the pressure of the cerebro-spinal fluid and reduce brain pulsation. A craniotomy (∼8 mm diameter) was performed over the right auditory cortex, and the dura was removed and replaced with a layer of 1.5% agar in 0.9% saline (see Fig. 1B). Cortical areas were identified by reference to landmarks on the skull, the blood vessel pattern, the presence and direction of a tonotopic gradient determined by preliminary recordings of multi-unit activity across linear electrode arrays (see Fig. 1C) and the relative sensitivity, response latencies and response type to noise or tones. We have previously used these criteria to define the cortical areas [21], [4], [22], [23], [9], [24].

#### Recording extracellular potentials

Recordings were made with glass insulated tungsten electrodes with tip lengths of 10–15 µm [25]. These were mounted as fixed linear arrays of between four and eight at a spacing of about 300 µm onto a circuit board that attached directly to a headstage amplifier (Medusa, Tucker-Davis Technologies, Alachua, Florida). These multi-electrodes were advanced by a piezoelectric motor (Burleigh Inchworm IW-700/710) in steps of 2.5 µm after an initial insertion of at least 150 µm. A range of stimuli were presented at each location to reduce the possible recording bias generated by playing only one search stimulus until a response was noted on any of the electrodes. If activity was located, the electrode position was fine tuned for better signal isolation. The search stimuli were either the 10 communication calls, 50 microsecond clicks, pure tones or white noise bursts (duration 100 ms) gated on and off with 8 ms cosine squared ramps and with a repetition period of 800 ms.

**Figure 3:**
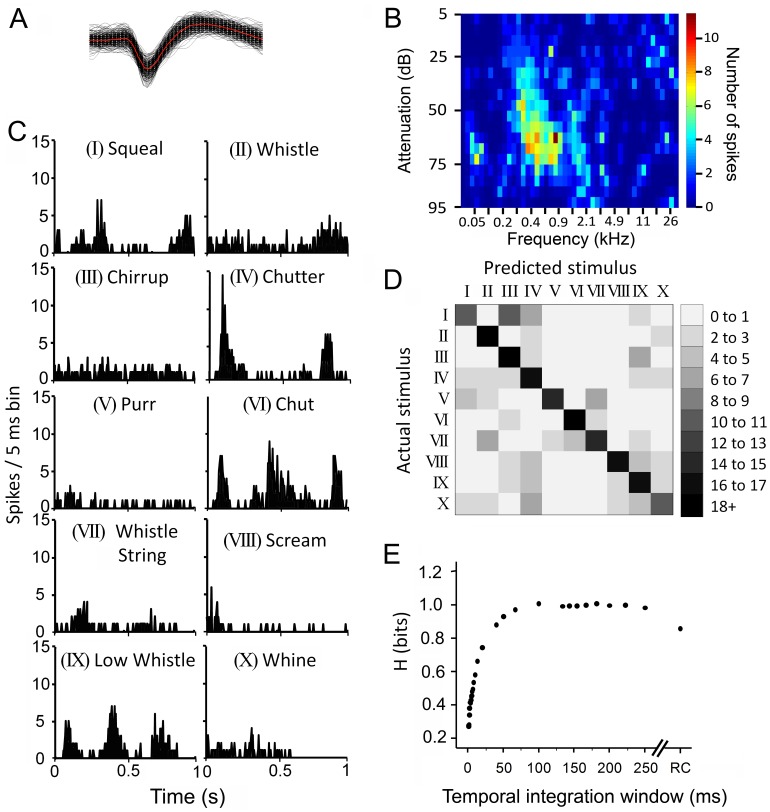
Spike distance metric example analysis. A. The spike sorted waveforms of a single neuron. The black lines are individual spikes and the red line is the average. **B.** The frequency response area. **C.** PSTHs of the responses of the neuron in A and B to all ten of the vocalizations. **D.** The confusion matrix for classification of the stimuli based upon the spike distance metric at an integration window of 8 ms. Note the greater number of counts along the diagonal, showing successful classification of the stimuli by the spike train. **E.** The information carried in the spike trains as a function of changes in the integration window.

Extracellular potentials were amplified and filtered (300–3000 Hz) and then digitized. Responses were collected using Brainware (v7.43, Jan Schnupp, Oxford University), and the recorded spikes were sorted using the Plexon Offline Sorter. Using the Plexon software to conduct a principle component analysis, clusters of spikes which had similar waveform properties were grouped together as belonging to a single unit. Statistical analysis was undertaken using Multivariate ANOVA followed by pairwise analysis to investigate whether the clusters differed significantly from one another and from the background noise. The sorted spikes (see inset in Fig. 3 and in Fig. S3) were then exported into Matlab for further analysis. The characteristic frequency (CF) and the minimum response threshold to pure tones were determined by making automated frequency/intensity plots (Figs. 1D and 3B). The 10 vocalizations were presented in a pseudo-random, interleaved pattern, and each was repeated 30 times. Responses to 30 repetitions of both broadband noise and clicks were also recorded. Auditory-responsive units were identified by an increase in firing rate to at least one stimulus: tone pip, noise burst, click or vocalization. Peristimulus time histograms (PSTHs) of the vocalization responses were plotted with 5 ms bins over a period of 2 or 3 s. PSTHs of the tone pips, noise and clicks were plotted within 5 ms bins for 300 ms.

### Data Analysis

Population responses within each cortical area were produced by summing the PSTHs of the responses of all neurons responsive to the various vocalizations. We included weak or borderline responses in these population responses by accepting responses that had as few as 8 spikes above the background rate, providing these were locked to the stimulus.

#### Correlation analysis

For the four calls that had a structure composed of rapidly repeating transient elements (chut, chutter, chirrup and purr: see Fig. 2 and Fig. S1), we initially correlated the population responses with the waveform envelope in the same way as described previously for the purr call [23]. However, the responses were better correlated with the first differential of the waveform envelope for the six calls that were more continuous (squeal, whistle, whistle string, low whistle, whine and scream: see Fig. 2). This provided a better fit with the response patterns than the envelope itself and was more likely to identify frequency transitions. For this reason, we correlated all population responses with the first differential to provide an appropriate comparison. Note, however, that the correlation values for the chut, chutter, chirrup and purr were lower to the first differential than to the envelope and hence represent a conservative estimate. The correlation function was calculated in Microsoft Excel™ according to the following formula:
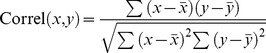
where x and y are the sample means average for the waveform envelope and the response PSTH. To allow for different response latencies, the PSTH was shifted by between 10 and 60 ms in 5 ms steps relative to stimulus onset and the largest correlation value (between −1 and +1) used as described previously [23]. Statistical comparisons were made using the SPSS statistics package (Apache Software Foundation).

Auditory-responsive units were identified by an increase in mean firing rate (2 standard deviations above base firing rate) or a peak in the PSTH (a peak was counted if it was two standard deviations above the base firing rate and had at least 12 spikes in a 5 ms time bin) to 30 repetitions of at least one stimulus: tone pip, noise burst, click or vocalization. We calculated both the mean stimulus evoked firing rate and the temporal characteristics of each response PSTH. The firing rate of the unit in response to each call was measured from the point at which the firing rate first exceeded two standard deviations above the spontaneous rate to the end of the call.

#### Call preference index

The call preference index for a neuron is the number of calls that evoked at least a half maximum firing rate in response to any call [26], [27]. This analysis was only applied to call responsive neurons.

#### Spike distance metric (SDM) analysis

A spike distance metric analysis [28], [29] was applied to the spike trains recorded from units in each area to assess the ability of each unit to classify the different calls as well as to determine the time scale at which the classification was best. A similar approach has been used to evaluate spatial tuning cues based on spike trains recorded from the inferior colliculus [30] and the auditory cortex [16]. The computation for a single neuron is illustrated in Fig. 3. Figure 3A shows the spike sorted waveform, 3B the response area and 3C the PSTHs in response to the 10 calls. The spike distance metric, or *Dspike*, between two spike trains is defined by the cumulative cost of steps required to transform one spike train into the other using either shifts of the timing of spikes or by adding or deleting spikes. Addition or removal of a spike has a cost of 1, while shifting spikes in time has a cost of *q*|Δ*t*|. The parameter *q* has units of s^−1^, and Δ*t* is the size of the time shift in seconds. Functions from the Spike Train Analysis Toolkit (a neuroinformatics resource provided by the National Institutes of Health Human Brain Project) were used to compute the *Dspike* distance metric for each neuron across a range of *q* values.

The ability for each neuron to accurately classify the different call stimuli was assessed by building a confusion matrix, *C*, from the *Dspike* values (Fig. 3D) [28], [29]. For each trial of a stimulus *α*, the average *Dspike* distance between that trial and those of spike trains in response to stimulus *β* was computed. The value of the confusion matrix for which the *Dspike* was, on average, minimal was then incremented by 1. This was repeated for all trials and stimuli. The information *H*, in bits, was then computed as:
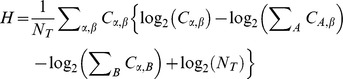
where *C* is the confusion matrix and *N_T_* is the total number of spike trains collected from the neuron [28]. This process was repeated for a range of cost values (*q*: 0, 8, 9, 10, 11, 12, 13, 14, 15, 20, 30, 40, 50, 75, 100, 150, 200, 250, 300, 350, 400, 450, 500, 600, 700, 800, 900, 1000, 1500, 2000, 2500) and the maximal amount of information about the stimulus set (as reflected by the information, *H*) and its associated temporal integration window was found (see Fig. 3E). Integration window values above about 1 s correspond to a purely spike rate-based code, while lower integration values correspond to an increasingly temporally-based code. When the movement cost for a given spike is greater than 2/*q* it is cheaper to add or delete a spike rather than move it, this results in temporal integration windows of 2/cost. Thus, a cost of 8 has a temporal integration window of 250 ms.

A lower bound for the classification information was determined by constructing a confusion matrix from data in which the spike trains were reassigned randomly to different stimulus categories. This eliminated any consistent relationship between the stimuli and the spike trains elicited by those stimuli.

## Results

### Response Classes

**Figure 4:**
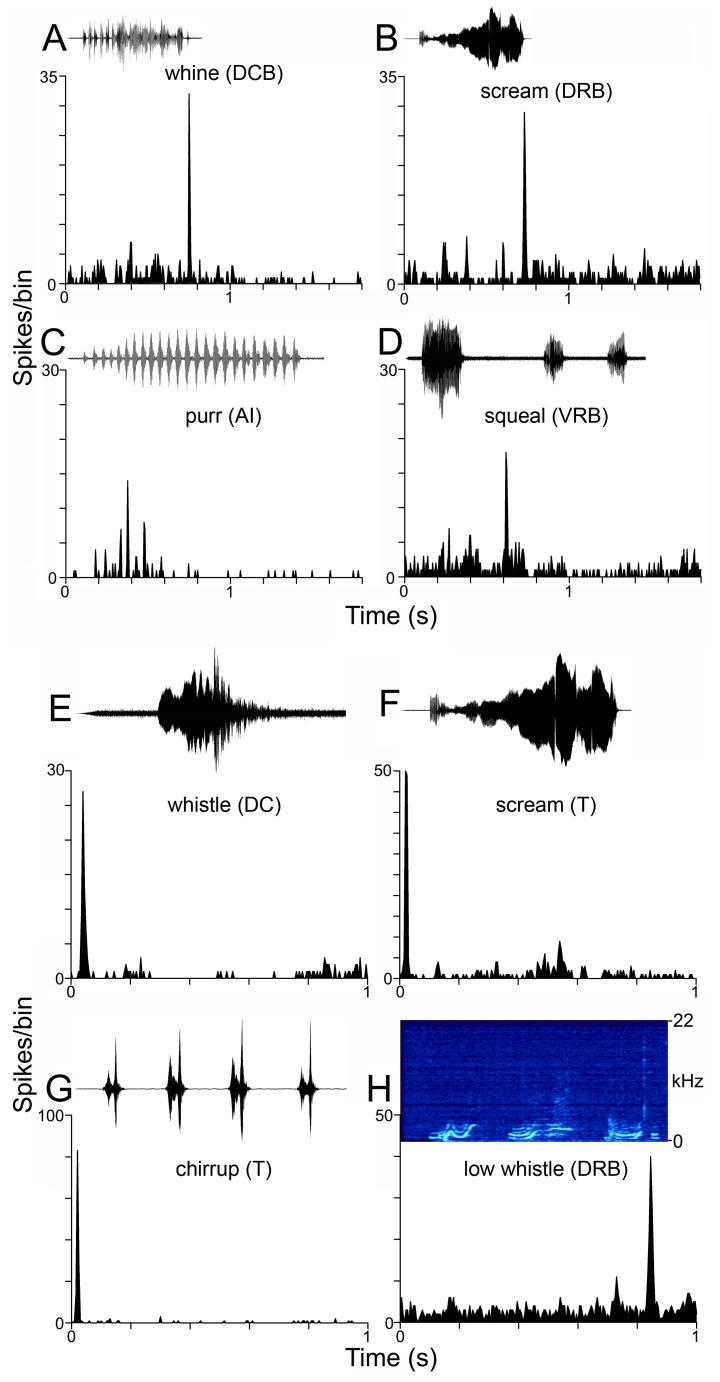
Responses to vocalizations that are neither onset nor isomorphic. Waveforms and PSTHs of the responses to various vocalizations. **A, B.** Offset responses. **C, D, H.** Responses during the vocalizations. **D, E, F.** No response to the vocalization *per se* but to the low level background noise in the recordings. **H.** The inset is the spectrogram of the low whistle on a timescale that allows the pronounced click, to which the neuron is responding, to be visualized.

Despite the fact that we used 10 call exemplars and recorded from 8 areas, most of the responses to vocalizations could still be fitted into four classes that are similar to those that have been previously described in the guinea pig thalamocortical system [31], [32], [23], [16]. For some neurons, the temporal pattern of the PSTH corresponded closely to the temporal structure of the call and had at least two peaks of increased firing, neither of which corresponded to the offset of the call. We refer to these as isomorphic because the best of them are closely related to the waveform envelope of the call (Fig. 2A). The second group includes onset responses which have a single peak in their firing rate close to the onset of the call (Fig. 2B). The third class includes cells that do not respond significantly to any of the main components of the call, but rather respond to clicks or changes in the background noise either during or preceding the call (Fig. 4). Some neurons (Figs. 4A, B) mainly give a single response at the stimulus offset, while others have peaks of activity within the call (Fig. 4C, D,H). The fourth group (Fig. 4E, F, G) includes cells that do not give any significant response to a vocalization. These are examples of neurons that respond much better to the onset of the background noise than to the vocalization itself. The background noise was relatively loud (∼30–40 dB SPL, ramped in over 25 ms) because some of the calls were initially low intensity, normalization amplified the background noise to a level that was suprathreshold for many neurons.

### Distribution of Sampled Units Across Each Area

We recorded from a total of 651 auditory responsive units from across the auditory cortex and sampled between 47 and 121 units from each area (see Table 1). We believe our sample of units represents the activity that is typical of each area. In each area that responded to tones, the range of CFs covered at least 4 octaves and recordings were made from at least three separate animals (Table 1). However, we did not systematically sample the whole isofrequency band in areas AI and DC, and these large areas may have contained subareas or columns that were not sampled.

**Table 1:** Responsiveness of units in each of the eight cortical areas to tones and calls.

Area	S	AI	VRB	VCB	DC	DRB	T	DCB
**Number tested**	**62**	**83**	**84**	**47**	**72**	**110**	**72**	**121**
**Mean** * **CF (kHz)**	8.5	3.6	4.4	–	10	6.4	11.8	14
**Range of CFs (kHz)**	0.2–30	0.1–22	0.2–12	–	0.5–33	0.3–33	1.4–34	1–33
**Mean and ^#^SD noise latency (ms)**	19.7(3)	15.4(5.1)	37.1 (17.6)	33.3(7.8)	15.7(4.3)	24.7 (14.4)	20.7 (10.3)	24.1(12.2)
**Mean and SD click latency (ms)**	17.9(3.6)	12.2(2.6)	26(14.8)	27.2 (6.6)	13.9(3.6)	13.8(3.3)	17(5.3)	20.7(8.4)
**Respond to call but not tone**	0%	9%	13%	79%	4%	1%	4%	11%
**Only respond to 1 call**	31%	12%	14%	30%	5%	16%	2%	19%
**Mean no. of calls responded to**	2.9	5	4.4	2.8	6.1	2.7	8.2	3.3
**Mean firing rate** **for calls. Spikes/s**	18.1	9.5	17.1	3.8	2.7	2.5	6.4	6.1
**Mean call correlation**	0.365	0.29	0.179	0.09	0.156	0.144	0.151	0.078
**Call representation**	sparse	dense	dense	varies	dense	sparse	dense	sparse

* CF, characteristic frequency; ^#^SD, standard deviation.

**Figure 5:**
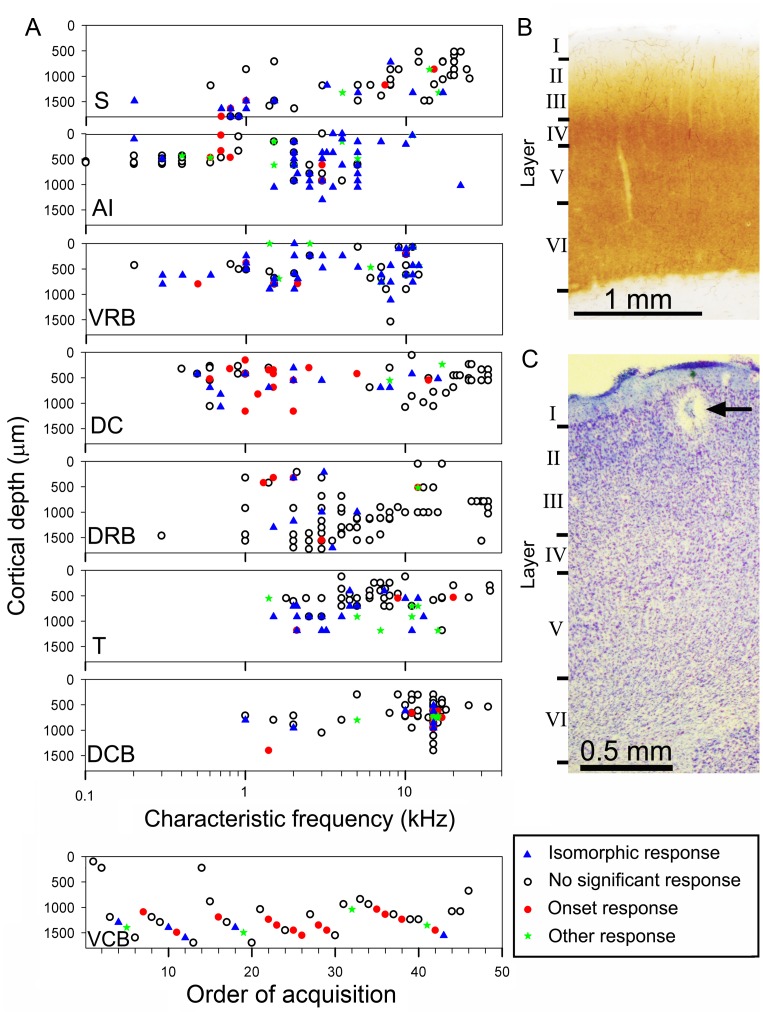
Distribution of call-responsive units in the eight auditory cortical areas. A. The recording depth and characteristic frequency (CF) of units that gave the same type of response to any calls that stimulated them. The three main types of response and non-response are shown by different symbols. Units in VCB usually did not respond to pure tones and we were unable to define a CF. Thus the VCB units were plotted in the order of their acquisition across seven tracks. **B.** Representative sections through the guinea pig cortex are shown on the right stained for cytochrome oxidase (upper panel) and **(C)** Nissl substance (lower panel). The lower panel shows a lesion (arrow) which we used in a previous study to relate electrode depth to cortical layer [33]. The cortical thickness was about 2 mm in the core area but it became thinner towards the belt areas and ranged from 2.2 to 1.6 mm.

We tried to avoid any laminar bias in our recordings by collecting units from whichever layer they were found in and recording from depths of between 100 and 1200 µm, which roughly corresponds to layers I – V [33], [24]. In some areas, we recorded from depths of 0–1600 µm, but in all areas except in VCB we mainly recorded from layers I – IV. In VCB we were unable to find many responsive units in the upper layers (Fig. 5). This was also true in area S despite the recording depths ranging from 500–1700 µm. Area S is located at the rostral edge of AI within the banks of the pseudosylvian groove. The presence of the groove causes the layers to dip down obliquely relative to the electrodes, so a nominal depth of 1600 µm may still correspond to layer IV [24].

#### (1) Proportions of cells responding to vocalizations and simple stimuli

The use of multiple search stimuli (noise, clicks, pure tones and communication calls) allowed us to sample cells which were responsive to only a subset of stimuli and to reduce the possible recording bias. They also allowed us to determine if there was any area that preferred complete vocalizations to the simpler sounds contained within them or *vice versa*. Fisher’s exact test was used to determine if an area had a greater or a lesser number of units that responded to a communication call than either noise, clicks or pure tones (Table 2). Area DRB and DC had significantly fewer units that responded to calls than to noise, clicks or pure tones. In addition, area DRB had significantly fewer units (25%) responding to communication calls than any other area (p<0.001 for all comparisons between areas).

By contrast, VRB had the highest proportion of call responsive units, and this percentage was significantly greater than that to noise (p<0.001) or clicks (p = 0.002). There were very few cortical cells that responded only to communication calls and not to any of the simple stimuli (13/651 cells). Although the majority of these call-selective cells were found in area VRB (VRB 10% 8/84, AI 4% 3/83, T 3% 2/72), they still only made up a small proportion of the cells recorded in that area. VCB had a higher proportion (89%) of units that were responsive to calls than to noise or tones, but this reflected the low number of units that responded to noise (68%) or tone pips (11%) rather than an unusually high proportion that responded to calls. Thus, although VRB showed evidence of preferring calls to simple stimuli, we were unable to find any evidence of a vocalization-selective area similar to the one described in the macaque monkey [3].

**Table 2:** Comparison of the responses to simple stimuli and calls in each of the cortical areas.

Area	S		AI		VRB			VCB		DC		DRB			T		DCB
**Number tested** **Total = 651**	**62**	**83**		**84**			**47**		**72**		**110**			**72**		**121**	
**Click (n)**	50	59		57			41		61		99			65		94	
**%**	81	71		68			87		85		90			90		78	
**Noise (n)**	55	62		51			32		65		50			66		93	
**%**	89	75		61			68		90		45			92		77	
**Pure tone (n)**	60	71		73			5		67		65			56		85	
**%**	97	86		87			11		93		59			78		70	
**Calls (n)**	48	67		77			42		49		27			57		94	
**%**	77	81		92			89		68		25			79		78	
**Proportions of cells responsive to communication calls in each cortical area compared with simple stimuli**																	
**Area**	S	AI				VRB	VCB		DC		DRB		T			DCB	
**Noise**						**	**		##		#						
**Click**						**			#		##						
**Tone**	##						**		##		##						

Fishers exact test compares the proportions of cells responsive to communication calls in each cortical area with the proportions that responded to simple stimuli (*more responsive to calls at the p<0.05 level, **more responsive to calls at the p<0.001 level, # less responsive to calls at the p<0.05 level, ## less responsive to calls at the p<0.001 level).

#### (2) Cells that only respond to one call

**Figure 6:**
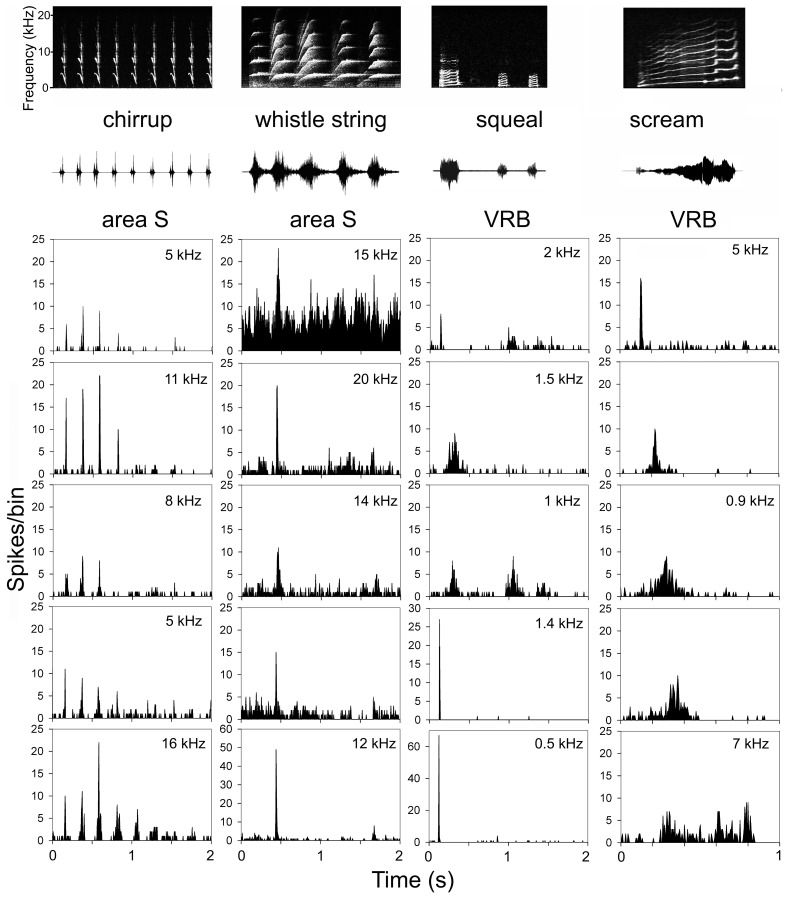
Examples of units that only responded to one out of the ten call exemplars. The PSTHs of five units that only responded to the individual call indicated, are shown in columns for areas S and VRB. The CF of the unit is given in the upper right corner of each panel if it was known. The spectrogram and waveform of the call used is shown at the top of each column.

Early studies in the primate brain looked for evidence of units that responded specifically to an individual call [15], [34]. We also studied this in the guinea pig. Individual neurons varied in the number of calls to which they responded with a few responding to all 10 calls, some not responding to any and the majority responding to a subset of the calls. As expected, some units were highly selective and only responded to one of the 10 exemplars presented (“unique responders” see Fig. S3). In area S, these unique responders usually gave reasonably strong isomorphic responses. This suggests that, rather than being so weakly excited by calls that only one response reached significance, these units are selectively excited by only one call. This is illustrated in Fig. 6, which shows the responses for 5 unique responders in areas S and VRB for four different calls.

Responses to each of the 10 calls were found in nearly all cortical areas, but two of the belt areas (area S and VCB) showed higher selectivity than the other areas when we measured the numbers of calls to which a unit would respond. Within these two areas, the mean number of calls to which a unit responded were lower (area S, 2.9; VCB, 2.8) and the proportions of units responding to only one of the calls were higher (area S, 31%; VCB 30%) than other areas (Table 1). Although area DRB units responded to an average of 2.7 calls, not many units responded to calls (25%), and the responses were typically weak. In all other areas, less than 20% of their units responded to only one call.

#### (3) Differences in the mean accuracy of a call’s temporal representation

When considering the responses to a call, it is important to study the degree to which a cell’s firing pattern gives an accurate representation of the call’s temporal structure. Examples of good isomorphic responses are shown in Fig. S2. The raster plots in this figure show the consistency of the responses which have a similar spike pattern for each call presentation. Isomorphic responses were found in all areas but were least common in areas DC, DCB and VCB (Fig. 5).

**Figure 7:**
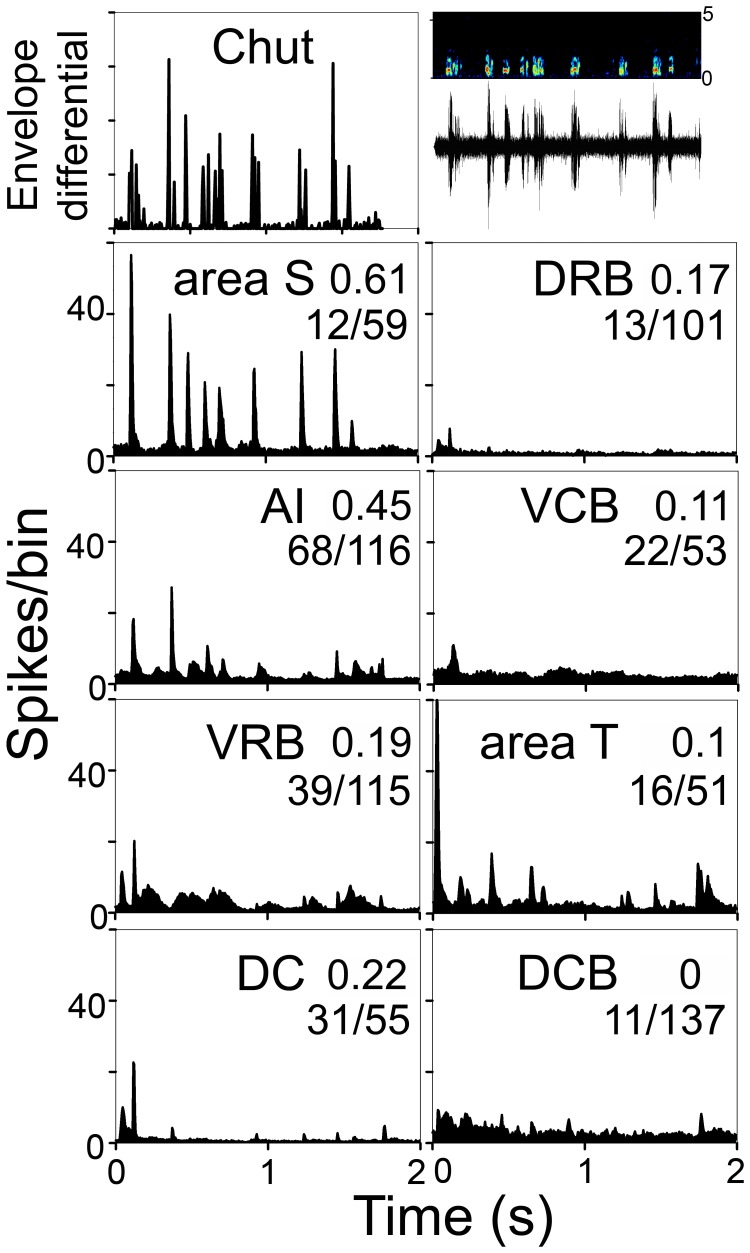
Population responses to the chut call in all eight auditory cortical areas. The top left panel shows the first differential of the waveform envelope of the chut (half-wave rectified) and the top right panel shows the spectrogram (0–5 kHz) and waveform. Most of the energy is in the range 0.25–2 kHz. The correlation between the first differential of the waveform envelope and the population response is shown at the right side of each panel. The number of units responding to the chut over the total number of auditory responsive units is shown as a ratio for each area. In area S 20% of units responded to the chut (12/59) and for the other areas the percentage responses are: AI 59%, VRB 34%, DC 56%, DRB 13%, VCB 42%, area T 31%, DCB 35%.

We used the population responses to study the distribution of the units with different response types. An isomorphic population response is evidence of a large proportion of responsive units within an area displaying an isomorphic response. The population responses for the chut call are shown in Fig. 7. The spectrogram, stimulus waveform and differential of the half-wave rectified envelope are shown at the top and the pooled responses for each of the eight cortical areas are shown below. The degree to which the responses followed the temporal structure of the call was quantified by measuring the correlation between the first differential of the waveform envelope and the PSTH (see Methods). These values are shown at the upper right of each panel for each area. Among the units that responded to the chut, those with the strongest isomorphic responses were in area S (correlation value of 0.61). AI had the next highest correlation (0.45), whereas areas DRB and the caudal belt mainly gave an onset response and had the lowest correlation values. There is a wide variation among areas in the percentage of units that responded to the stimulus, with 20% in area S 59% in AI and DC, VRB and area T ranging in between.

**Figure 8:**
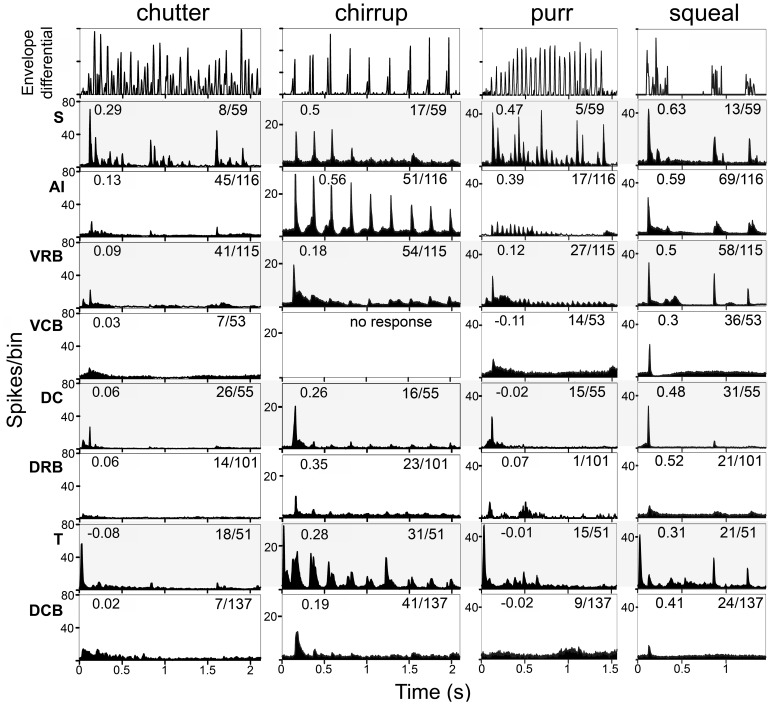
Population responses to four calls by units in each of the eight cortical areas. The top panel of each column shows the first differential of the waveform envelope. Each subsequent row shows the responses from one cortical area as indicated on the left. The numbers on the left of the response panels show the correlation between the mean population response and the first differential of the envelope in the top panel. The numbers on the right show the proportion of recorded units in each area that responded to the call by an increased firing rate and that were used in forming the population response.

**Figure 9:**
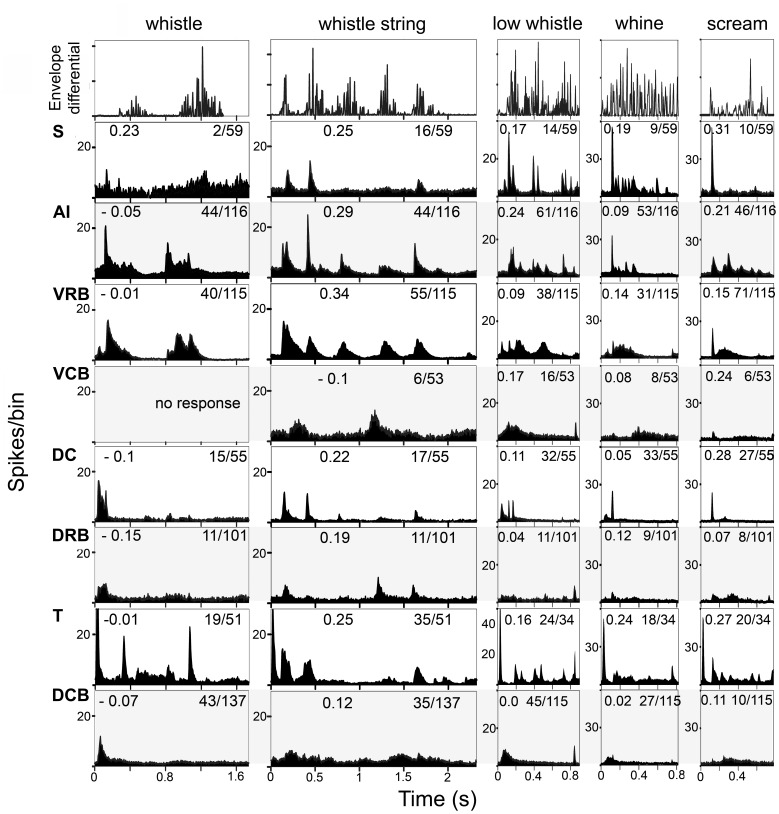
Population responses to five calls by units in each of the eight cortical areas. The top panel in each column shows the differential of the waveform envelope. Each row of panels show the responses for one cortical area as indicated on the left. The numbers in the panels give the correlation values (left) and proportion of units that responded to the call (right).

The population responses to the other 9 calls are shown in Figs. 8 and 9. The spectrogram and waveform for each of these calls are shown in Fig. 2 and Figure S1. In Fig. 8 and 9, the top panel shows the first differential of the waveform envelope. The number of responsive units as a proportion of all units that respond to auditory stimuli is shown on the right of each panel.

Comparison of the population responses indicates that the most highly correlated responses are usually located in area S (highest mean values for 7 calls), but these responsive cells are sparsely distributed. By contrast, areas AI and VRB have a denser representation of responsive cells, but their mean correlation values are not as high as in area S (see Table 1). The other five areas have lower mean correlation values, and all areas except area T have most (at least 66%) of their responses only to the onset of the call.

#### (4) Discrimination among communication calls using a rate code

In the marmoset a rate code may also be important in representing vocalizations [18], [35]. Romanski and others [36] had previously shown that 17% of primate ventrolateral prefrontal cortex neurons are highly selective for communication calls, responding to one call with at least double the firing rate of any other call. We measured the mean firing rate of cortical neurons evoked throughout the duration of each call. We then compared the firing rates for all the calls to which a unit responded in order to judge the ability of a unit to differentiate between calls through a change in firing rate.

**Figure 10:**
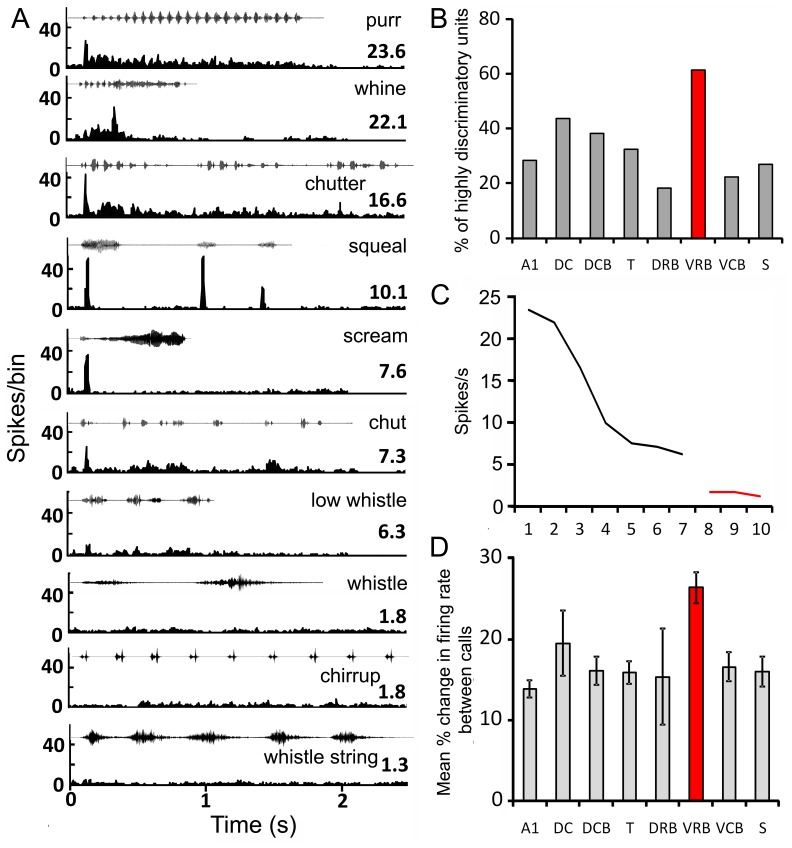
Comparison of changes in firing rate for responses to different calls in different areas. A PSTHs of the responses by a single unit to each of the 10 calls. The waveform of each call is shown above its respective PSTH and the firing rate over the duration of the call (spikes/s) is shown at the right hand side. The responses are arranged in order of firing rate with the highest at the top. The responses to the bottom three calls were not significantly above the background firing rate. **B** Histogram showing the percentage of responsive units in each area that gave responses that had firing rates that were at least twice as high for one call as for any others. VRB (red bar) had a higher proportion of these highly discriminatory units (61%) than any other area. **C** Graph showing the change in the firing rate between different calls for the unit illustrated in panel A. The slopes for the calls where there was no response are shown in red. **D** By taking the overall slopes of units such as that shown in panel C it is possible to calculate the mean % change in firing rate between calls for all units that respond to two or more calls. When these values were plotted for each area VRB was the area with the steepest changes in firing rate (red bar) and a reasonably small variance as indicated by the error bars.

Units within the auditory cortex as a whole showed a high degree of preference for particular calls, with a large number of units responding with more than double the firing rate to one call than for any other call (Fig. 10B). Striking in these data are the large number of highly discriminatory units in VRB, where 61% of call responsive units had a firing rate for one call that was at least twice as high as for any other call (Fig. 10B).

We tested to see if units that responded to multiple calls were giving a graded response to different calls. If so, then one unit (or more likely, a population of such units) could be used to discriminate among several calls using a rate code. The mean percentage change in firing rate was compared for all the calls to which a unit responded. Within each unit, responses were rank ordered from the maximum to the minimum (e.g. Fig. 10A), and the average percentage change in firing rate between responses to different calls was compared (e.g. Fig. 10C). By comparing the average of these values between cortical areas, we could test whether some areas were modulating firing rates in response to calls more than other areas (Fig. 10D). A one-way ANOVA revealed a main effect of area on the average percentage change in firing rate (F (DF = 7) = 5.84, p<0.001). Sheffe *post hoc* analysis revealed that VRB units displayed, on average, a significantly greater degree of modulation of firing rate between responses than AI (p<0.001), DCB (p = 0.008) and T (p = 0.011), while differences in VRB were marginally greater than S (p = 0.055) and VCB (p = 0.07). Differences were not significantly greater than those in DC (p = 0.764) or DRB (p = 0.511), though this is more likely to reflect the high variance in these areas resulting from the few call responsive units rather than suggesting these areas are as good as VRB at modulating firing rate in response to calls.

**Figure 11:**
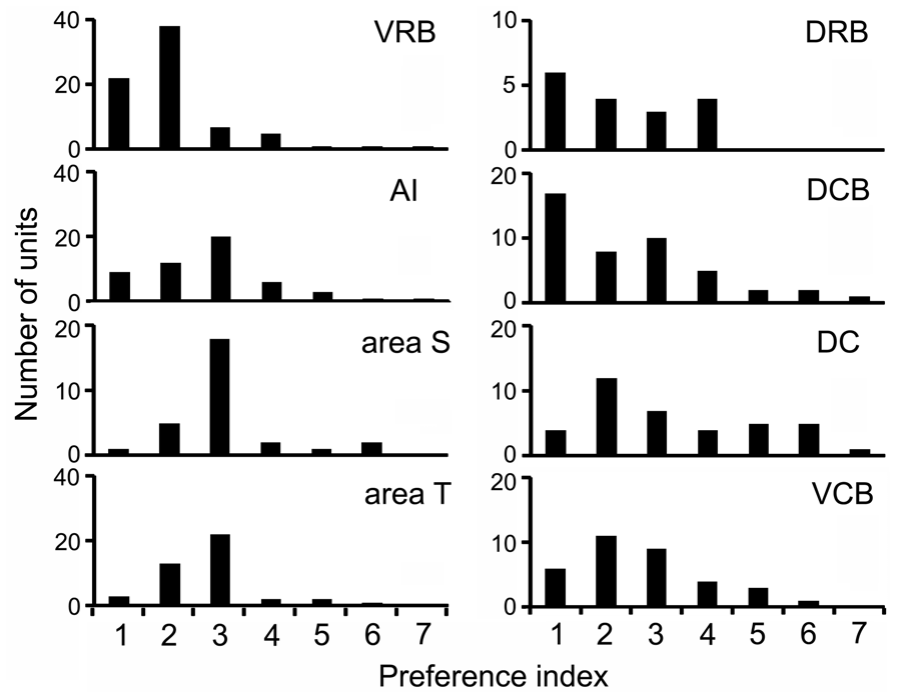
Call selectivity in different cortical areas. Call preference index (see methods) responses to different calls in different cortical areas.

Although VRB units typically responded to many calls, the call preference index (Fig. 11) indicated that they discriminated well among those calls using a rate code. VRB units were more selective on the call preference index than units from the other areas, while units from AI, T and S showed greater selectivity than units from DC or the other belt areas. These data, along with the large proportion of call responsive units in VRB, suggest that VRB is highly involved in processing communication calls and may use a rate code to discriminate among them.

### Spike Distance Metric Analyses

**Figure 12:**
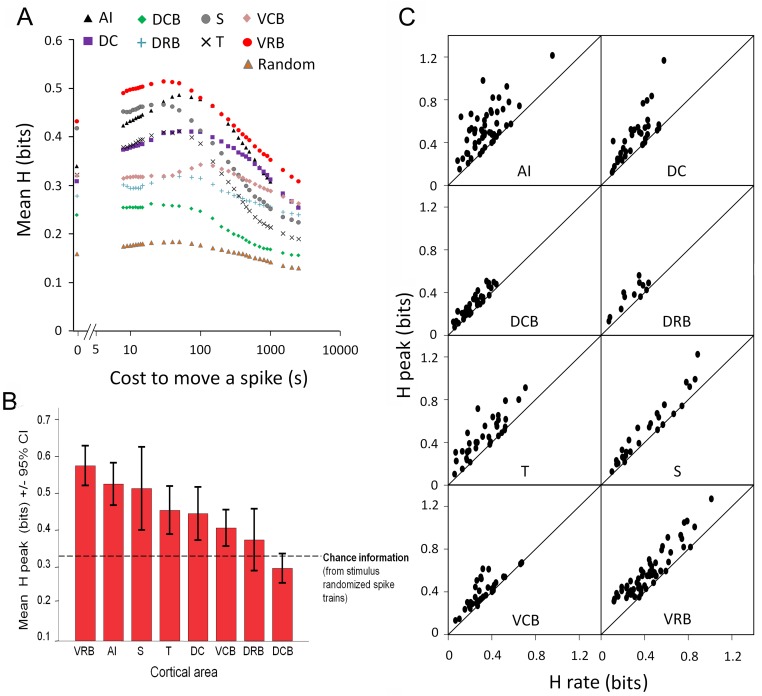
Spike distance metric analysis. **A**. Average value of the information about the vocalizations in the activity of populations of neurons in the different cortical areas (see key) as a function of the cost of moving a spike (see methods). **B**. Rank ordered maximum information from the different cortical areas. **C**. Peak information as a function of the information at zero cost to move a spike (i.e. the information carried in a spike rate code alone). Values above the diagonal line of equality indicate additional information carried in the temporal patterning of the spikes.

The above analyses consider rate and temporal aspects of the responses independently. At least at one level, this is clearly simplistic. Combining the magnitude of the response with the pattern of response may provide more flexibility in the manner of representation and hence potentially may provide more discriminability [37]. We used the SDM to evaluate the mutual information about the calls carried by units in the different cortical areas and also to assess the degree to which the information is carried by a rate or a temporal code. The results of these analyses, across all cortical areas, are shown in Fig. 12. In Fig. 12A the mean information in each of the cortical areas as a function of the cost is shown. It is clear from this figure that the peak cost value does not occur at zero in any cortical area. This indicates that no cortical area uses a purely rate code for vocalizations. This is reinforced in Figure 12C, in which the value at cost of zero (*H* rate: the information in a rate code alone) is plotted against the value at the peak for different units in each cortical area. All points on the diagonal indicate units that are using a purely rate code. In most areas, some neurons utilize a rate code while many other neurons carry additional information about the vocalizations in their temporal firing pattern. The greatest vocalization discriminating information (*H* peak) was typically recovered at an integration window of 40 ms. Within all eight cortical areas, 50% of the *H* peak values were recovered within the integration windows of between 13 and 67 ms. Figure 12B shows the rank ordered mean of the peak amount of information in the different cortical areas. It is immediately apparent that VRB, AI and S carry the most information about the vocalizations, the three belt areas VCB, DRB and DCB are close to chance performance and DC and T are a little better than chance.

## Discussion

### Specialized Cortical Areas for Processing Vocalizations

Acoustic communication is common across many invertebrate and anuran species [38], as well as songbirds and other animals whose territories would have overlapped those of early mammals. These heterospecific calls along with conspecific calls may have contributed to the evolution of multiple auditory cortical areas. All mammals studied so far have multiple auditory cortical fields, and there is compelling evidence that the various areas may have very different functions [2]. Some auditory cortical areas in the macaque monkey are thought to have different roles in processing communication calls [27], [39,], and there is a voice-selective area in the anterior temporal lobe of the macaque [3]. In other mammalian species, however, it is not clear whether any areas have a specific role in processing conspecific vocalizations or even what criteria should be used in assessing this. This is in contrast to songbirds where there are specialized sensorimotor nuclei that are involved in the production and analysis of conspecific songs [40]. There is already some evidence for differences in call processing by the different auditory areas in the guinea pig [8], [9], [41], but in this study we conducted a more systematic analysis. We found no evidence for an area that was predominantly involved in processing vocalizations. The core area AI and the belt areas VRB and area S showed evidence of being more involved in processing vocalizations than any of the other cortical areas, but they all also responded well to simpler stimuli such as noise bursts or tones. Even VRB only contained 10% of units that responded to vocalizations but not to pure tones, clicks or noise bursts. Our previous work on VRB showed that units there also responded well to other relatively simple stimuli such as amplitude modulated tones [21].

Recent studies of mammalian cortical processing are increasingly using awake preparations as they provide a more natural brain state than deep anesthesia [16], [42], [43]. The dramatic effect of anesthetics on processing vocalizations has been shown in studies of the songbird forebrain nucleus HVc. Most neurons that respond to the bird’s own song in the anesthetised or slow-wave sleep state do not respond to the same song when the bird is awake [44], [45]. In the guinea pig surgical doses of an anesthetic will often suppress the neural response to a particular call but may also enhance it [46]. We plan to make recordings from awake animals in future. Nevertheless, in this study we wanted to directly compare our results to other studies of the guinea pig, which were mainly performed under surgical anesthesia [31], [32], [16], [41], [47]–[50]. Anesthesia eliminates concerns that can be present in awake recording, such as the sleep/wakefulness cycle [51], [52] or the role of attention [53], and in the guinea pig the responses in an awake animal do not appear to be radically different to those anesthetised with urethane [16]. Furthermore, vocalizations can provide an emotionally charged stimulus that evokes a physical reaction from an animal and makes it difficult to hold the same unit for very long. Thus, we decided that it was best to use anesthesia for this initial comparison between all the areas. Our study relies on the untested assumption that the anesthetics have the same effects on sensory responses in all the cortical areas. This is a weakness that could only be addressed by making comparative recordings in awake animals.

### Significance of Isomorphic Responses

One of the more striking findings in this and previous studies [32], [23], [16] of the guinea pig auditory cortex was the presence of isomorphic responses where neurons responded to a call with a high firing rate and a spike pattern that often had a precise temporal correlation with the call envelope. They were very different from the more common types, which did not respond to the call or only gave an onset response. The contrast between these types of units was reminiscent of the contrast between the cells of the cat primary visual cortex when they were stimulated by a line as opposed to a small circle [54]. It is possible that interconnected groups of neurons with these isomorphic responses form networks specialized for processing vocalizations [55] and that these networks are mainly spread across three cortical areas rather than a single vocalization-specific area. Isomorphic responses were found in all cortical areas but were much more common in the areas AI, VRB and the small field (S). It would be useful to know if the isomorphic responses were organized into functional columns that spread across all cortical layers and are thought to be a basic processing unit of most cortical areas [56]. Our current study was not designed to answer this question, but our previous work in the guinea pig [7] indicated that there may be columns in low-frequency (<1.2 kHz) AI. Low-frequency AI contains cells that show isomorphic responses to one or a number of calls with a similar degree of accuracy to those in the inferior colliculus and thalamus [57], [47], [48]. These isomorphic responses were certainly present in the output layers of AI: both layers II and V/VI [46], [33], [13]. Adjacent to these columns in low-frequency AI were others that did not respond or only gave onset responses [7], and the correlation values for most units in AI were not very high. This is consistent with earlier studies showing that many cortical units gave responses that did not accurately reflect the waveform envelope. This was shown in ferret [58], songbird [59]–[61] and guinea pig [16]. There have not been any studies of either area S or VRB to show whether or not they contain columns. However, area S is approximately the same size as a macrocolumn in the primate visual cortex [54] and could be arranged into mini columns, each of which might have distinctive responses to one or more calls. Unfortunately, our data was collected in a way that did not allow us to address this question.

### Selectivity Versus Discrimination

Two alternative ways in which a neuron could be specialized for call responses is by 1) responding to many of the calls but having graded responses where the number of spikes generated was significantly different between calls (a rate code), or 2) being very selective and only responding to one out of the 10 calls. The second strategy is just an extreme form of the first, but many neurons in VRB used the first strategy while more neurons in area S used the second. VRB was exceptionally good at discrimination among calls using a rate code, with the majority (61%) of call responsive units in this area responding to one call with at least twice the firing rate than for any other call. This high level of response rate modulation was greater than that of any other cortical area, including AI. VRB also carried more information about the vocalizations than any other area. Higher order areas in the macaque, specialized for processing communication calls, have also been shown to discriminate among communication calls using a rate code [27], [3], [36].

In contrast to VRB, area S had the highest proportion of units that only responded to one call (31%). Some of these unique responders had a high precision (correlation values of >0.6 for chut). This selectivity may be due partly to simple spectrotemporal filtering properties. For example, none of the units in area S that responded uniquely to the chirrup responded to tones of less than 2 kHz, while all of the unique responders to purr in AI responded to tones with a CF centred on 250 Hz. However, we did not have sufficient numbers of each type of unique responder to establish any clear relationship to pure tone responses. In addition, we suspect that the so called “unique” responders may have responded to more than one call if a range of exemplars had been used for each call. We have already shown that some units in AI could have very different responsiveness for different exemplars of the short purr [50]. Counting “unique” responders was a convenient way of assessing the specificity of a neuron’s response but was not evidence that it would only ever respond to that particular call. A high degree of call specificity has also been described in the squirrel monkey cortex [15], [34] as well as the macaque, where specificity was measured by a simple call index based on sensitivity to a selection of seven calls [27]. These results are not thought to represent evidence for high level “grandmother” cells but rather show a low level combination sensitivity for vocalizations [43]. Studies in primates have failed to detect “grandmother” cells in either primary [62]–[64], [55] or secondary areas of auditory cortex [42].

### Comparison of Belt Areas

Area VRB was the area where units carried the greatest call discriminatory information. It receives cortical inputs from AI and area T [22], but its thalamic input and projection targets have not been studied. It would be interesting to map the projections of the VRB to see if it has projections to areas known to be involved in vocal communication such as the amygdala [65]. Areas S also had units that carried a relatively high amount of information about the calls. It is known to project rostrally [22], but it is not known if this projection is to a distinct prefrontal area or directly to the part of the anterior cingulate cortex that elicits vocalizations when stimulated electrically [66]. In the gerbil, Budinger et al. [67] have shown projections directly from the auditory cortex into the anterior cingulate cortex.

The third belt area with significant levels of call information is area T. In this study the calls were presented at too high a sound level to properly assess its responses. We have previously shown that area T is particularly sensitive to the tooth chatter call [41] and gives an accurate time-locked response to the individual tooth clicks of the call over a 40 dB range of sound levels. Presenting the tooth chatter at a sound level 40 dB below the level used in this study abolished the onset response to the noise background while retaining the strength of response to the call itself. Caudal belt areas DCB and VCB and belt area DRB contained very little information about the calls and may not have an important role in processing vocalizations.

### Hierarchical Processing of Communication Calls

In the macaque, areas specialized for processing vocalizations have been described in the anterolateral belt area [27], superior temporal region [3] and prefrontal cortex [36], and all may form part of an anterior call discrimination pathway [68]. Vocalization selective units have also been described in the caudal insula of the macaque monkey [69] and may form part of a separate processing pathway. Evidence for separate processing pathways has previously been obtained in the guinea pig auditory region in electrophysiological [7] and imaging studies [5]. Although the imaging study did not identify area S, a more recent imaging study indicated a separate tonotopic area that appears to correspond to area S [70]. The sensitivity of areas AI, VRB, T and S to conspecific vocalizations could simply represent a preference of these areas for spectro-temporally complex signals. It would therefore be interesting to test the sensitivity of these areas to heterospecific vocalizations.

These findings are consistent with the proposal that caudal areas may be part of a pathway that is more involved in processing sound localization [71] and suggest that the dorsal pathways are not involved in processing vocalizations. There is no evidence of a parabelt in the guinea pig [12], [4], and it is not known if there is a guinea pig homologue of the medial prefrontal areas described in the macaque monkey [36]. The arrangement of auditory areas in the guinea pig seems to be different from other rodents, and homologies with the auditory areas in the primate brain are difficult to draw [1]. Despite these limitations, studies of guinea pig call processing may be relevant to studying the human brain because of the spectral range of their vocalizations [11] and the basic neural mechanisms involved [49].

## Supporting Information

Figure S1 **Spectrograms of the vocalizations.** The time base of each spectrogram has been optimized to allow different features of the various vocalizations to be visualized.
(TIF)

Figure S2 **Unique responder.** PSTHs of the response of a single unit in area S with a CF of 11 kHz to the 10 different vocalizations. The inset shows the spike sorted action potentials. This unit was highly selective and only responded to the chirrup.
(TIF)

Figure S3 **Units can respond faithfully to the multiple phrases of repetitive calls.** Examples of multi-units, from four cortical areas, showing the consistency of their response to each phrase of a repetitive call over 20 repetitions. In each panel the top trace shows the waveform of the call, the middle trace shows a raster plot of spike times and the bottom trace shows a PSTH of the number of spikes in each 5 ms time bin. The CFs of the units vary over a large range and are as follows: **A** 3 kHz, **B** 9 kHz, **C** 0.8 kHz, **D** 17 kHz.
(TIF)
