# Correction: Processing of Communication Calls in Guinea Pig Auditory Cortex

**DOI:** 10.1371/annotation/3b354af8-3468-4d8e-84bd-d3245dfa198c

**Published:** 2013-05-24

**Authors:** Jasmine M. S. Grimsley, Sharad J. Shanbhag, Alan R. Palmer, Mark N. Wallace

The following information was missing from the Funding Statement:

While working in Ohio JMSG and SJS were supported by research grant R01 DC00937 (JJW) from the National Institute on Deafness and Other Communication Disorders of the U.S. Public Health Service. 

