# Supplementary figures and images for: Processing of Communication Calls in Guinea Pig Auditory Cortex
Source: PLoS One. 2012 Dec 12;7(12):e51646. doi: 10.1371/journal.pone.0051646 (PMC3520958; doi:10.1371/journal.pone.0051646)

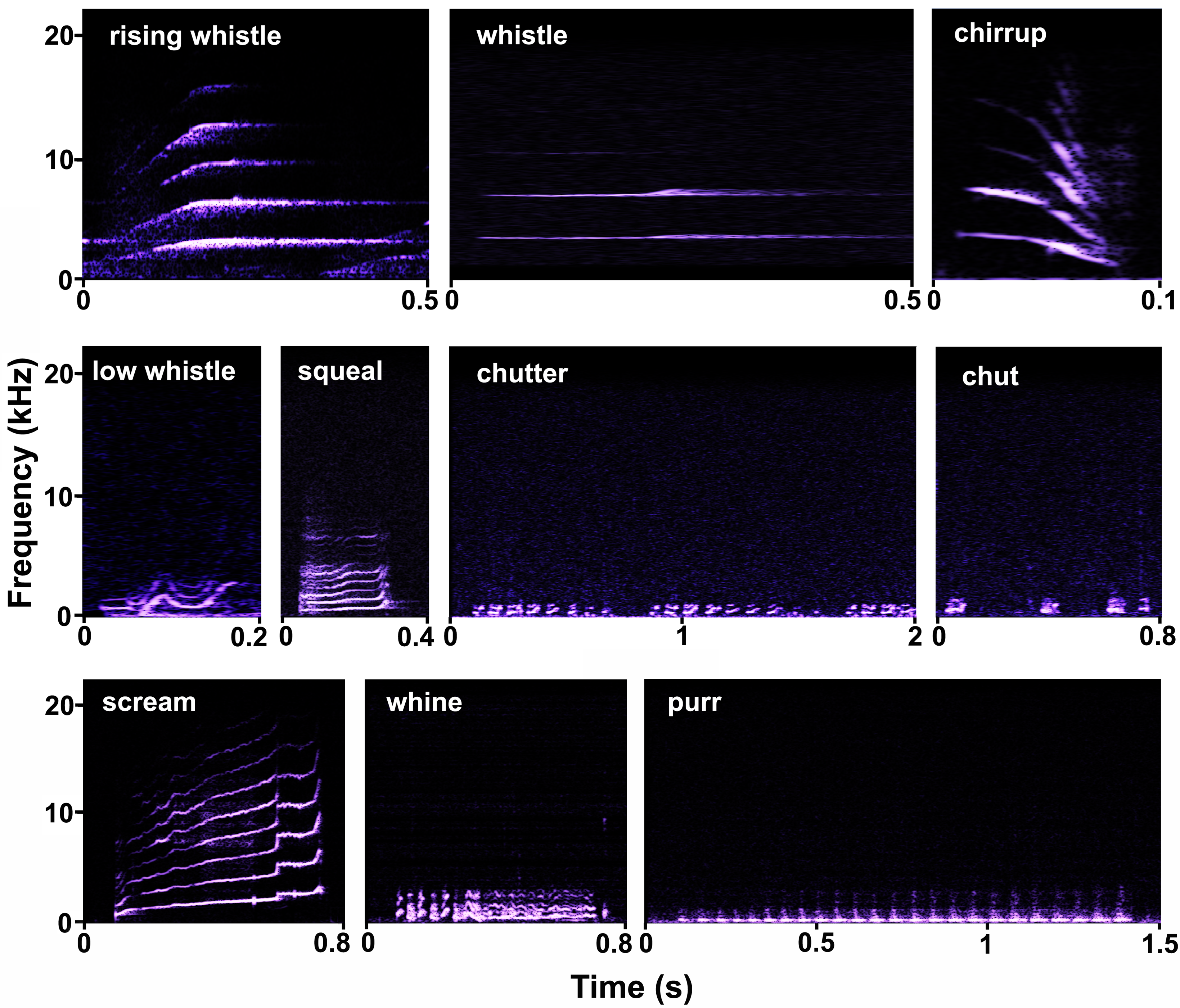

Supplement: Figure S1 — Spectrograms of the vocalizations. The time base of each spectrogram has been optimized to allow different features of the various vocalizations to be visualized. (TIF) [file pone.0051646.s001.tif]

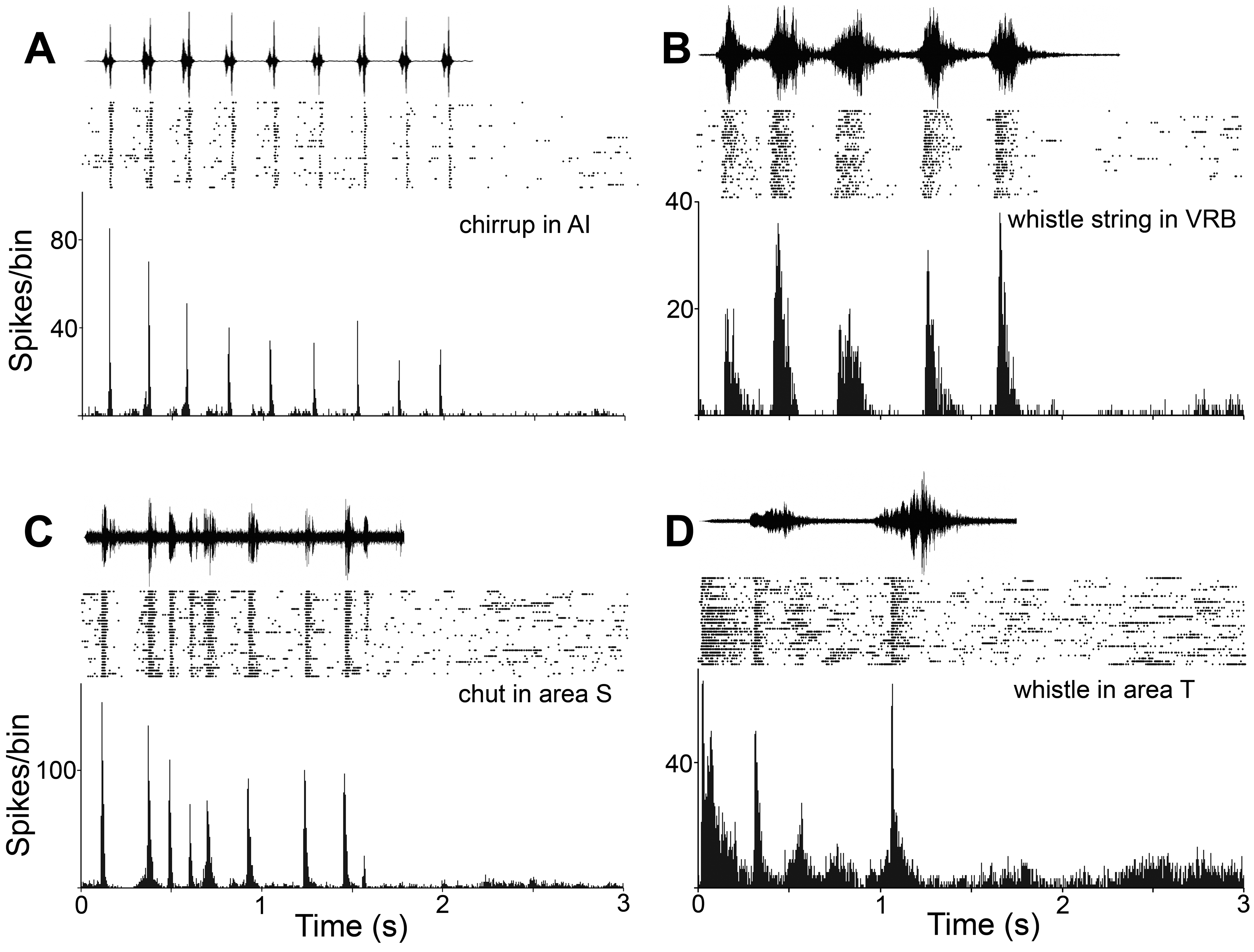

Supplement: Figure S2 — Unique responder. PSTHs of the response of a single unit in area S with a CF of 11 kHz to the 10 different vocalizations. The inset shows the spike sorted action potentials. This unit was highly selective and only responded to the chirrup. (TIF) [file pone.0051646.s002.tif]

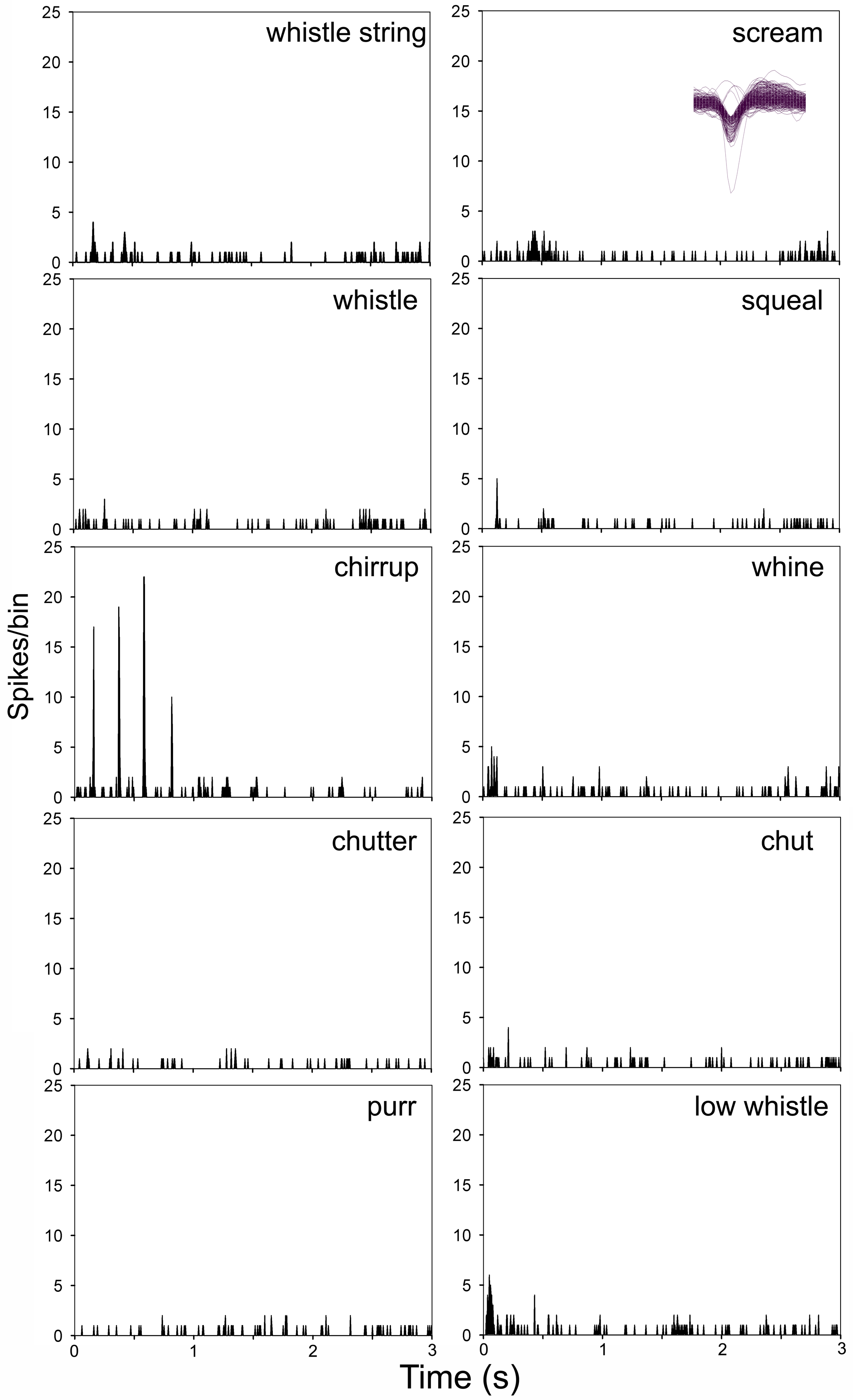

Supplement: Figure S3 — Units can respond faithfully to the multiple phrases of repetitive calls. Examples of multi-units, from four cortical areas, showing the consistency of their response to each phrase of a repetitive call over 20 repetitions. In each panel the top trace shows the waveform of the call, the middle trace shows a raster plot of spike times and the bottom trace shows a PSTH of the number of spikes in each 5 ms time bin. The CFs of the units vary over a large range and are as follows: A 3 kHz, B 9 kHz, C 0.8 kHz, D 17 kHz. (TIF) [file pone.0051646.s003.tif]
